# Rapid diagnosis of *Mycoplasma pneumonia* infection by denaturation bubble-mediated strand exchange amplification: comparison with LAMP and real-time PCR

**DOI:** 10.1038/s41598-018-36751-z

**Published:** 2019-01-29

**Authors:** Wenqiang Shi, Manman Wei, Qing Wang, Hongwei Wang, Cuiping Ma, Chao Shi

**Affiliations:** 1 College of Life Sciences, Qingdao University, Qingdao, 266071 PR China; 2 Shandong Provincial Key Laboratory of Biochemical Engineering, College of Marine Science and Biological Engineering, Qingdao University of Science and Technology, Qingdao, 266042 PR China; 3 The Clinical Laboratory Department of the Affiliated Hospital of Qingdao University, Qingdao, 266101 PR China

## Abstract

*M*. *pneumoniae* infection is often ignored due to its similar clinical symptom with respiratory tract infections caused by bacteria or viruses, and thus leading to misdiagnosis and delayed treatment. It is critical to develop a rapid, sensitive and specific diagnosis method. Denaturation Bubble-mediated Strand Exchange Amplification (SEA) was established, which is an isothermal method with only a primer pair and one *Bst* DNA polymerase. Notably, colorimetric SEA assay was developed with simple visual readout, making instrument-independent in detection step. The method could detect as low as 1.0 × 10^4^ copies/mL genomic DNA within 60 min. Considering that more than 80% infected patients have 1.0 × 10^5^−1.0 × 10^7^ copies/mL *M*. *pneumonia* DNA, SEA is available for the practical diagnosis of *M*. *pneumoniae* in clinical specimens. Through comparing 224 sputum specimens, excellent performance of SEA assay with 90.48% sensitivity and 100% specificity relative to real-time PCR was observed. Compared with LAMP, a comparable sensitivity and low false positive rate was observed for SEA method. Therefore, SEA is a promising method for detecting *M*. *pneumoniae* directly from clinical specimens, which is especially suitable for point-of-care testing in primary care facilities and resource-limited settings with minimal equipment and technological expertises.

## Introduction

*Mycoplasma pneumoniae*, one of common pathogens of respiratory tract infection, is frequent cause of approximately 10–30% community-acquired pneumonias (CAP) in adults and children^1^. Significantly, *M*. *pneumoniae* can lead to up to 40% CAP among school-aged children from 5 to 15 years olds^2^. *M*. *pneumoniae* infection is not only the primary cause of atypical pneumonia but also usually causes extrapulmonary complications, such as nervous system disorder, encephalitis, myocarditis and asthma etc.^2^. The outbreak of *M*. *pneumoniae* epidemic often occurs in 3 to 7 years cycles and may last up to 2 years each time^3^. During pneumonia epidemic periods occurred in 2011–2013 worldwide^4^, up to 28% and 30% of *M*. *pneumoniae* positive respiratory tract samples were reported in Germany^5^ and China^6^, respectively. The peak incidence reported in Scotland and Finland was 14.2 and 14.5 per 1000 population CAP^4^. However, *M*. *pneumoniae* infection in children is often ignored due to its similar clinical symptom with respiratory tract infections caused by bacterial or viral infection, and thus leading to misdiagnosis and delayed treatment. Consequently, early, rapid and sensitive diagnosis of *M*. *pneumoniae* is critical to estimate and prevent large-scale outbreaks of CAP caused by *M*. *pneumoniae*.

Nucleic acid amplification techniques are currently considered a promising alternative method for rapid diagnosis of *M*. *pneumoniae* infections, showing high specificity and sensitivity compared to conventional cultivation methods^7^. Real-time PCR has been widely applied to detect *M*. *pneumoniae* clinically, which is more sensitive and do not require agarose gel electrophoresis; However, this method requires the complex operation, specialized instruments and trained personnel, and thus is time-consuming and high cost^8^. In recent years, a series of isothermal nucleic acid amplification techniques become more powerful alternative for point of care diagnosis, which can amplify nucleic acids at a single temperature and read out results using either real time fluorescent probes or colorimetric methods^9^. Among them, loop-mediated isothermal amplification (LAMP) is proven to have nearly the same sensitivity and specificity for detecting *M*. *pneumoniae* as PCR^10^, and thus is a useful technique for the rapid diagnosis of *M*. *pneumoniae* infection in clinical practice^11^. However, LAMP assay requires at least four primers targeting six specific regions, making primer design more complex; it is also highly susceptible to carryover contamination, leading to false positive results^12^. Recently, a novel isothermal amplification method, denaturation bubble-mediated strand exchange amplification (SEA) has been developed, which requires only a simple primer pair and utilizes successive natural strand “breathing” to form unwound double-stranded DNA rather than heat denaturation^13^. In general, dsDNA can dynamically dissociate due to ambient thermal fluctuations, even without the variation of temperature or pH, producing a single-stranded denaturation bubble^14^. Then, a short oligonucleotide primer could invade the denaturation bubble, triggering the extension by DNA polymerase to generate the amplification products. Considering that denaturation step is not required, the amplification can be performed at a single temperature below the melting temperature of dsDNA, eliminating the dependence on thermal cycler. More importantly, one pair of primers and one *Bst* DNA polymerase could trigger SEA reaction, simplifying reaction system. Considering the above advantages, SEA technology became another promising option for simple, rapid and sensitive diagnostic detection.

In this study, we established a SEA method for rapid detection of *M*. *pneumoniae* and compared it with LAMP method and real-time PCR. The objective is to provide a rapid and simple detection method for early diagnosis of *M*. *pneumoniae* infection.

## Results and Discussion

### SEA assay for *M*. *pneumonia*

**Figure 1:**
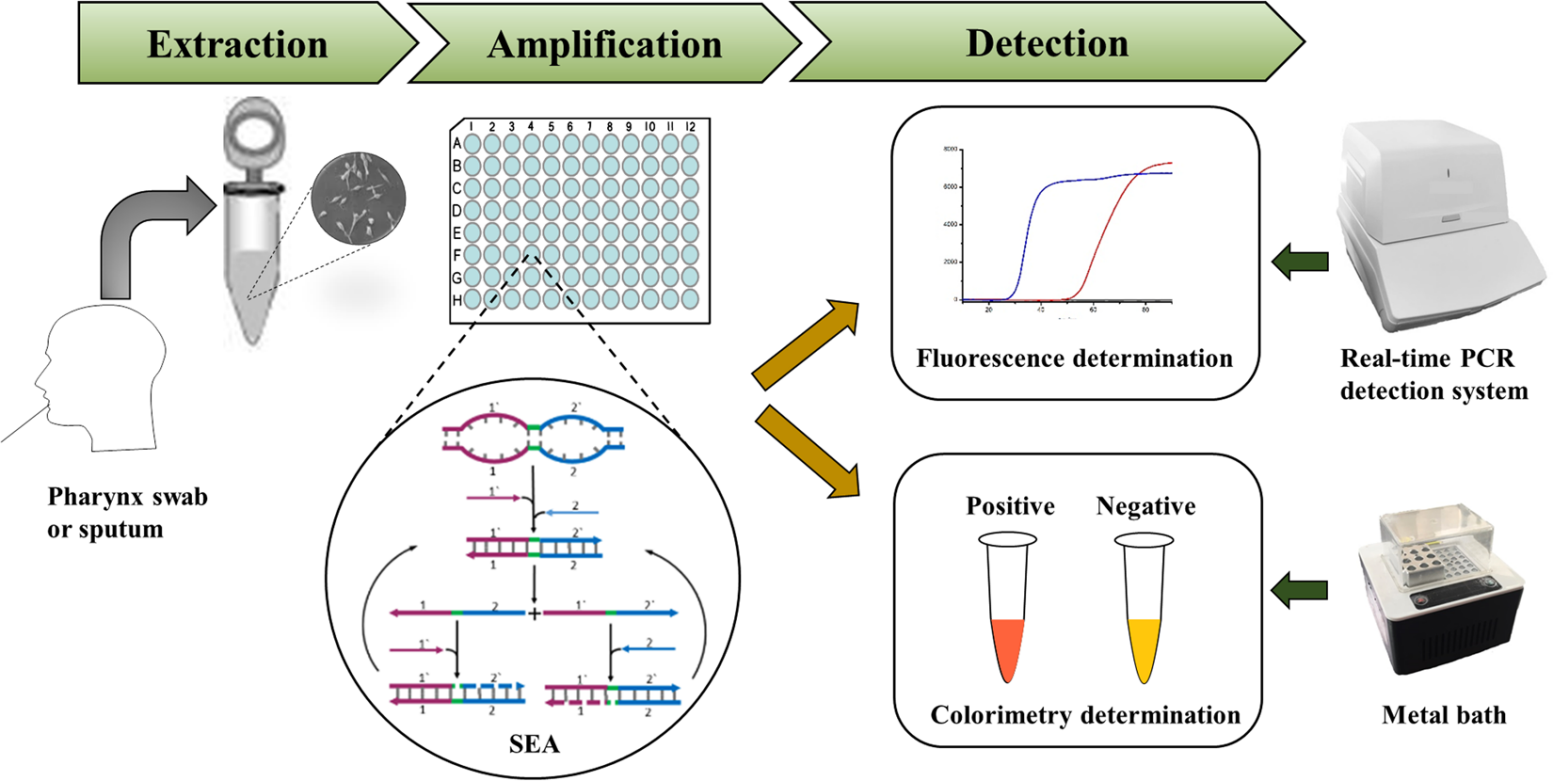
Schematic illustration of SEA assay for *M*. *pneumoniae* infection in clinical specimens.

SEA assay has been accepted as a simple and rapid isothermal detection method since the first report in 2016^13,15^. SEA reaction requires only a pair of primers and targets a short sequence with 40–60 bp length, and thus it is easy to find a unique sequence as amplification target for a variety of pathogenic bacteria. Generally speaking, SEA assay has a promising application for clinical, food and environmental analysis. The workflow for the detecting of *M*. *pneumoniae* by SEA was shown in Fig. 1. The extracted genomic DNA from bacteria strains or sputum samples was used as the template for SEA reaction. The amplification was either fluorescently monitored or visually observed by colorimetric analysis. During the colorimetric detection, the color of positive reactions changed from orange to purple red, while negative reaction remains orange. Therefore, SEA assay provides a simple, rapid and specific method for detecting *M*. *pneumoniae*, which was suitable for on-site and rapid diagnosis.

**Figure 2:**
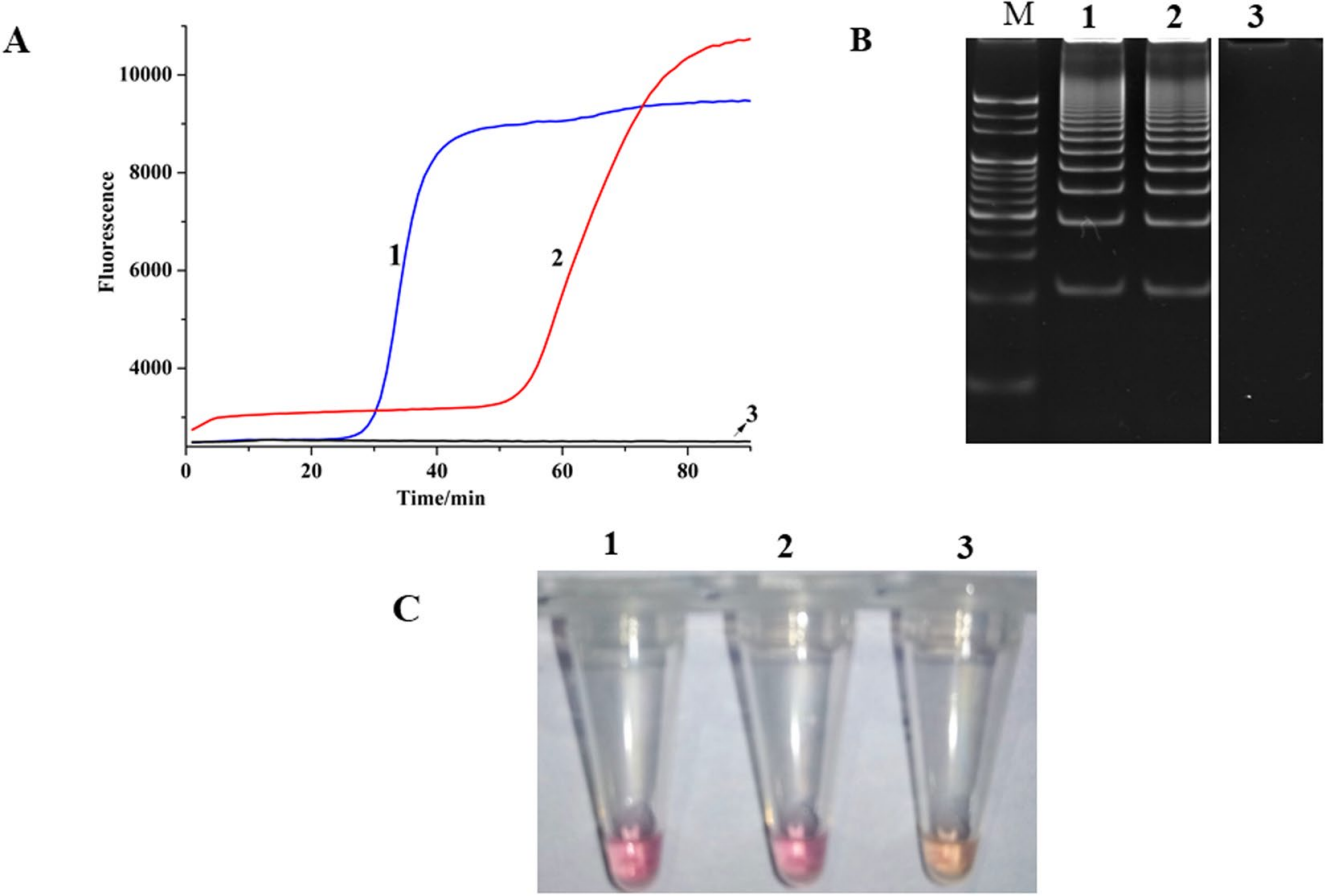
The feasibility of SEA to detect *M*. *pneumoniae*. (**A**) The real-time fluorescence curves. (**B**) Native PAGE of the corresponding amplification products (Supplementary Fig. S3. Lane 1, lane 2 and lane 6) (**C**) Colorimetric SEA assay using a pH sensitive dye as the indicator. 1 represented the vector plasmid DNA containing 16S rDNA sequence of *M*. *pneumoniae* M129, 2 represented genomic DNA extracted from sputum specimen infected by *M*. *pneumoniae*, 3 represented no template control, and M represented the 20-bp ladder marker (TaKaRa).

The feasibility of SEA was firstly evaluated by detecting a vector plasmid DNA containing 16S rDNA sequence of *M*. *pneumoniae* M129 which served as a positive control and a sputum specimen infected by *M*. *pneumoniae* (Fig. 2). As expected, the significant fluorescence signals in the reactions of the positive control and the sputum specimen compared with the no-template control (NTC) indicated that SEA could effectively detect *M*. *pneumoniae* (Fig. 2A), which was further verified by the expected 43 bp amplification products in native PAGE (Fig. 2B). These results demonstrated the feasibility and suitability of SEA for *M*. *pneumoniae* detection from clinical specimens. More importantly, SEA assay results could be directly observed by pre-adding colorimetric visual dye, by which positive reaction would be confirmed by color change from orange to purple red (Fig. 2C). As shown in Fig. 2C, the positive reactions developed a purple red color while reaction for NTC retained its orange color. Obviously, colorimetric SEA assay enabled result readout to realize the direct observation by the naked eye, providing a better and easier-to-understand visualization without a need for sophisticated instruments, such as fluorescence detector and gel electrophoresis imager. Therefore, SEA amplification is possible when only a simple water bath is used to maintain the isothermal conditions, making it especially suitable for point-of-care testing (POCT) in resource-limited settings in developing countries.

### Sensitivity and Specificity of SEA to detect *M*. *pneumoniae*

The specificity of SEA to detect *M*. *pneumoniae* was evaluated using 11 respiratory near neighbors (e.g. *Streptococcus pneumoniae* and *Chlamydia pneumoniae*) or other pathogens as listed in Materials and Methods. The fluorescence signal increase was only observed in amplification reaction containing DNA template from *M*. *pneumoniae*, whereas all other pathogens displayed no fluorescence signal increase (Supplementary Fig. S1). Therefore, no cross-reactivity was found between *M*. *pneumoniae* and those tested pathogens, suggesting the good specificity of *M*. *pneumoniae* detection, which is particularly important for the differentiation of *M*. *pneumoniae* from other pathogens leading to respiratory tract infection.

**Figure 3:**
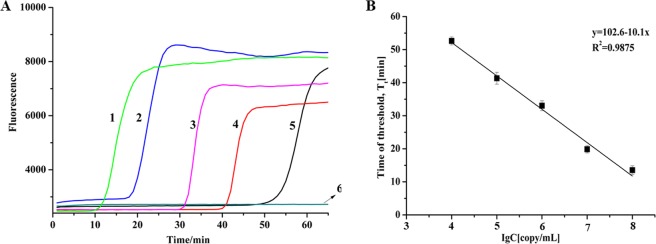
The sensitivity of SEA to detect *M*. *pneumoniae*. (**A**) The real time fluorescence curves with target sequence concentration of 1.0 × 10^8^ (1), 1.0 × 10^7^ (2), 1.0 × 10^6^ (3), 1.0 × 10^5^ (4), 1.0 × 10^4^ (5) and 0 (6) copies/mL. (**B**) The linear relationship between the threshold time (T_t_) value and the logarithm of target sequence concentration (copies/mL).

To determine the sensitivity of SEA for detection of *M*. *pneumoniae*, serial 10-fold dilutions of plasmid DNA with target sequences ranging from 1.0 × 10^4^ to 1.0 × 10^8^ copies/mL were used as a template. As shown in Fig. 3A, the significant fluorescence signal increase was observed for the reaction with different concentration of target sequence. The minimum amount of DNA with real time fluorescence signal was 1.0 × 10^4^ copies/mL within 60 min and colorimetric result were shown in Supplementary Fig. S5. The linear relationship was obtained between the threshold time value (T_t_) and the concentration of target DNA ranging from 1.0 × 10^4^ to 1.0 × 10^8^ copies/mL (Fig. 3B), yielding a corresponding correlation coefficient (R^2^) of 0.9875, which demonstrated the great stability of SEA assay to detect *M*. *pneumoniae*. Compared with the reported 2.2 × 10^3^ copies/mL for LAMP assay and 1.1 × 10^3^ copies/mL for real-time PCR^16,17^, a lower sensitivity was obtained for SEA reaction of *M*. *pneumoniae* detection. However, high sensitivity of LAMP assay often means high possibilities in false positive problem due to aerosol pollution^18^. As reported previously, LAMP assay was highly susceptibility to aerosol pollution, thus leading to false positive problem^18^. The false positive problem for LAMP assay was also found in the *M*. *pneumoniae* detection of our clinical specimens as discussed in section 3.4. In this study, SEA assay of *M*. *pneumoniae* could be completed within 60 min, showing that it is a time-efficient method.

Previous studies reported that *M*. *pneumoniae* carrier rates of 0.1–13.5% were detected in healthy individuals^3^. Asymptomatic carriage of *M*. *pneumoniae* must be considered when detecting *M*. *pneumoniae* infection in clinical specimens using amplification-based methods. Thus, it is crucial to establish a reliable diagnosis approach for *M*. *pneumoniae* infection which can distinguish real pathogens from asymptomatic carriage. A threshold of 10^4^ copies/mL genomic DNA was proposed to distinguish clinical infection for *M*. *pneumoniae* from carriage^19^. However, the true prevalence of asymptomatic carriage of *M*. *pneumoniae* in healthy individuals is unknown, so no threshold value is generally accepted. In a previous report, authors analyzed the copy numbers of *M*. *pneumoniae* DNA in sputum specimens from children with *M*. *pneumoniae* infection^20^. The results demonstrated that 103 of 120 children with *M*. *pneumoniae* infection have 1.0 × 10^5^ –1.0 × 10^7^ copies/mL genomic DNA, with a proportion as high as 85.53%. Therefore, the higher copy number than 1.0 × 10^5^ copies/mL genomic DNA in sputum specimens generally means real clinical infection for *M*. *pneumoniae*. Considering that the established method could detect as low as 1.0 × 10^4^ copies/mL genomic DNA, SEA assay is available for the diagnosis of *M*. *pneumoniae* infection in practice application.

### SEA assay for *M. pneumoniae* infection in clinical specimens versus real-time PCR

In order to evaluate the effectiveness of our SEA method in clinical practice for POCT, a total of 224 sputum specimens from upper respiratory tract infection patients were tested with SEA assay. As shown in Table 1, 38 in 224 samples were diagnosed with *M*. *pneumoniae* infection, showing a positive proportion of 16.96%. Of these specimens, 42 were known to be *M*. *pneumoniae* positive based on prior testing by real-time PCR, whereas only 38 of these specimens were identified as *M*. *pneumoniae* positive with SEA assay. This result demonstrated that SEA assay yielded a sensitivity of 90.5%, compared to real-time PCR as a reference. The Ct values of four positive specimens detected by real-time PCR but negative by SEA assay were 35.02, 30.2 30.88 and 37.20 respectively and the sequencing results were perfectly matched with our target sequence, further confirming real-time PCR assay results (Supplementary Fig. S4). In contrast, all 182 specimens with negative real-time PCR results were identified as *M*. *pneumoniae* negative with SEA assay, which corresponded to a specificity of 100.0%. To the best of our knowledge, this is the first study to evaluate SEA assay as a rapid diagnostic method for *M*. *pneumoniae* infection. Although SEA assay is only 90.5% as sensitive as real-time PCR, 100.0% specificity demonstrated the reliability of detection results. In this study, 4 specimens were tested with real-time PCR-positive but SEA-negative results, leading to a low sensitivity of SEA assay compared with “gold standard” real-time PCR. There were two possible reasons for this result. Firstly, this result may be attributed to low *M*. *pneumoniae* load in these sputum specimens, which cannot be successfully detected by SEA assay. In addition, we could not exclude the possibility that these real-time PCR-positive specimens were actually innocent bystanders rather than real pathogens. Although SEA assay only showed the 90.5% sensitivity compared with real-time PCR, 100.0% specificity demonstrated that this method supports for clinical diagnosis with *M*. *pneumoniae* infection. More importantly, SEA assay had a low possibility for false-positive amplification. Therefore, SEA assay had a high performance for *M*. *pneumoniae* detection in clinical specimens.

**Table 1:** Comparison of *M*. *pneumoniae* detection by SEA, real-time PCR and LAMP directly from clinical sputum specimens.

SEA result	Real-Time PCR		LAMP		Total
	Positive (n = 42)	Negative (n = 182)	Positive (n = 41)	Negative (n = 183)	
Positive	38 (90.48)	0 (0)	36 (87.80)	2 (1.09)	38 (16.96)
Negative	4 (9.52)	182 (100)	5 (12.20)	181 (98.91)	186 (83.04)
Total	42 (18.75)	182 (81.25)	41 (18.30)	183 (81.70)	224 (100)

Data were presented as number and proportion (%) of specimens.

**Figure 4:**
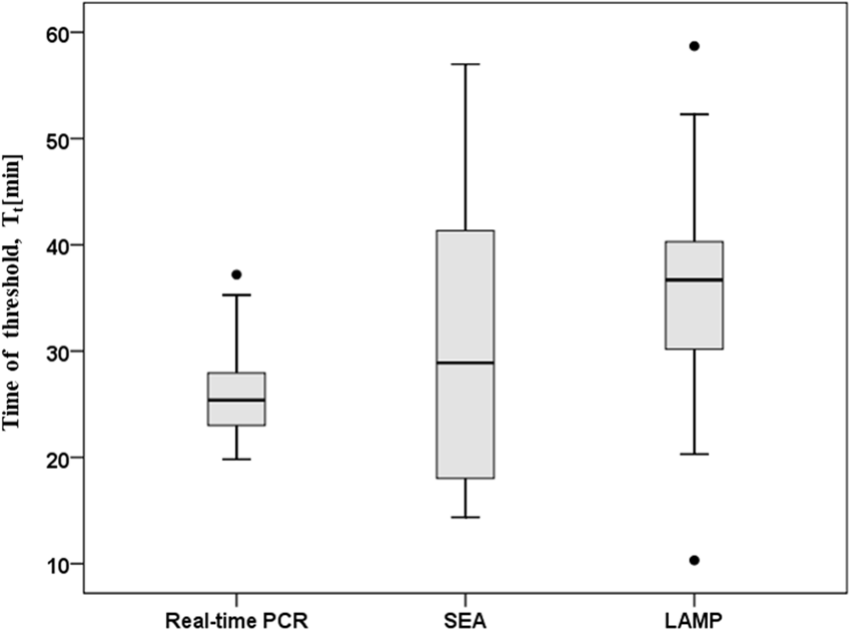
Distribution of threshold time (T_t_) values of *M*. *pneumoniae* detection in clinical samples by SEA, LAMP and real-time PCR assay. For real-time PCR, T_t_ value was the corresponding time of cycle threshold (C_t_). The Box-plot contained the mean, inter quartile range, non-outlier range, and outlier.

The threshold time (T_t_) values obtained for SEA positive samples were compared with that of real-time PCR (Fig. 4). For real-time PCR, T_t_ value is the corresponding time of cycle threshold (C_t_). The T_t_ values obtained for SEA showed high variability with a wide distribution range of T_t_ values ranging from 14.36 to 52.21 min. SEA assay for *M*. *pneumoniae* detection in clinical samples took about 19 min – 60 min, while the run time of real-time PCR was about 23 min – 50 min with the C_t_ values ranging from 20.5 to 35.27 min. Although T_t_ values were significantly higher for SEA assay than real-time PCR (P = 0.046), SEA assay enabled rapid detection of *M*. *pneumoniae* from clinical specimens within 60 min, showing a median T_t_ value of 30.5 min. In this study, real-time PCR were performed using a commercially *M*. *pneumoniae* diagnostic Kit (Sansure Biotech Inc., Changsha, Hunan, China), which used a simplified reaction procedure with only 15 s denaturation and 30 s step for annealing and extension, resulting in a faster runtime compared to typical real-time PCR procedure. In practice, the existing real-time PCR assay for *M*. *pneumonia* are not standardized and may differ in the run time ranging between 20 min to 60 min^21^. The detection time for SEA method established in this study is comparable to some reports for *M*. *pneumonia* diagnostic using real-time PCR^22^. However, real-time PCR method requires an expensive thermal cycler, hindering its wide application as a point-of-care diagnostic, especially in resource-limited settings.

### Comparison of SEA method with LAMP

SEA assay for *M*. *pneumoniae* was also compared with LAMP method, which has been reported to be a useful isothermal detection method for *M*. *pneumoniae* with high sensitivity^23^. Of 224 sputum samples, 41 (18.30%) were positive in the LAMP assay, which is higher than that of 38 (16.96%) positive samples detected by SEA assay (Table 1). However, two of positive samples detected by “gold standard” real-time PCR were still positive in SEA assay but not in LAMP, implying false negative results for LAMP assay. Additionally, five of the specimens were negative in SEA assay but positive in LAMP, showing a low sensitivity of SEA method than LAMP with the values of 90.5% and 95.23% compared to real-time PCR, respectively. Of these five, one positive sample in LAMP was negative in both SEA and real-time PCR, implying the possibility of false positive. Although a comparable or higher sensitivity of LAMP with real-time PCR was also reported in other studies for *M*. *pneumoniae* detection^16,24^, LAMP method failed to detect 2 real-time PCR positive samples, resulting in an analytical specificity of 98.91%. Thus, it seems SEA assay showed a higher specificity than LAMP method when detecting *M*. *pneumoniae* in real clinical specimens.

LAMP reaction is widely accepted to have greater or comparable sensitivity than real-time PCR due to its amplification mode by four or six primers^25^. The LAMP assay has been reported to detect *M*. *pneumoniae* as low as 200 copies/mL genomic DNA^24^ and has been commercialized for *M*. *pneumoniae* diagnostic in Japan and China considering its high sensitivity, cost-effective and thermal cycler independence^25^. In practice, a high proportion of false-positive amplification has been reported for LAMP method and must be extensively retested to confirm specificity^16^. In LAMP reaction, false positive result usually occurs due to carryover contamination, wherein high sensitivity makes re-amplification easy to occur using amplicons from previous LAMP reactions as templates^26^. Another reason for false positive problem is LAMP-like amplification independent of template, which is driven by self-priming of generated stem-loops after LAMP autocycling is triggered^26^. In a previous study, about 5.9% false positive rate was reported for LAMP detection of *M*. *pneumoniae* directly from respiratory clinical specimens^16^. In this study, one specimen with LAMP positive result but SEA and real-time PCR negative result were confirmed to be false positive by PAGE gel electrophoresis (Supplementary Fig. S2). Obviously, fluorescent signal and amplification product were observed for LAMP reaction but not for SEA assay. Colorimetric assay for LAMP and SEA reaction also confirmed the concordant result (Supplementary Fig. S2). Therefore, LAMP method produced a 2.4% false positive rate herein while SEA assay showed no false positive result. When compared T_t_ values, no significant difference was found between SEA and LAMP for *M*. *pneumoniae* diagnostic from clinical specimens (p = 0.121), showing the median T_t_ values of 30.5 and 35.1 min, respectively. LAMP method completed the reaction in 14 min – 65 min, corresponding to T_t_ values ranging from 10.3 min to 58.7 min (Fig. 4). Thus, the run time of SEA was comparable for LAMP method when detecting *M*. *pneumoniae* from clinical specimens. In summary, SEA method was a promising isothermal method, which possesses not only LAMP-similar advantage, such as reaction at constant temperature and independence of sophisticated instruments, but also possesses low false positive and high specificity compared with LAMP method.

Compared with real-time PCR and LAMP, the most prominent advantage of SEA is its low cost due to simple reaction system and low requirements for instruments and professional personnel. The total costs for real-time PCR, LAMP and SEA assay were estimated based on labor costs and prices for reagents, supplies, and equipment maintenance. The regent cost of each run for real-time PCR and LAMP was estimated to be €2.8 and €8.4 respectively, whereas it only costs 28 cents for SEA regents. Labor and instrument costs would be higher for real-time PCR because it requires an expensive thermal cycler and trained professionals. In summary, SEA method is more cost-effective compared with real-time PCR and LAMP.

Considering that extracted clinical specimens frequently contain substances that inhibit enzyme based nucleic acid amplification processes, negative amplification test results do not necessarily indicate absence of infection^27^. It is of significant to introduce an internal control during practical *M*. *pneumoniae* diagnosis of clinical specimens. An internal control can help to identify inhibitory specimens by monitoring amplification of a second target nucleic acid. The negative result for the primary target could be validated by successful amplification of the internal control. For real-time PCR and LAMP, an internal control could be introduced, either by multiplexing and distinguishing between target and internal control amplifications (e.g. using probes with different reporter dyes) or by normalizing target Cts to the Cts of reference sequences that are spiked into the sample. Theoretically, introducing an initial control in SEA assay could be achieved by distinguishing amplicon annealing temperature (Tm) or threshold time value (Tt) from target sequence. Unfortunately, it is not possible to distinguish reference and target sequences in colorimetric SEA reaction. There is still a challenge to introduce an internal control in colorimetric SEA reaction.

In conclusion, the SEA method for rapid detection of *M*. *pneumoniae* from clinical specimens was established and compared with real-time PCR and LAMP. The SEA assay completed the reaction within 60 min. More importantly, colorimetric SEA assay further simplified readout procedure and enabled it suitable for point-of-care testing. Additionally, SEA assays had lower levels of false positive contamination relative to the LAMP assays and are more likely to be effective in resource-limited settings for improved pathogen detection. In summary, the SEA assay will enable rapid, low-cost and visual detection of *M*. *pneumonia* and have promising application potential for earlier recognition of outbreaks, especially in resource-limited settings.

## Methods

### Bacterial strains and clinical specimens

A total of 11 reference strains including *Streptococcus pneumoniae* R6 (ATCC BAA-255), *Klebsiella pneumonia subsp. pneumoniae* (ATCC 13883), *Haemophilus influenza* (ATCC 51907), *Chlamydia pneumoniae* strain AO3 (ATCC VR-1452), *Staphylococcus aureus* subsp. *Rosenbach* (ATCC 6538), *Escherichia coli* O157:H7 Strain EDL933 (ATCC 43895), *Stenotrophomonas maltophilia* YF-6 (ATCC 17807), *Mycobacterium tuberculosis* H37Rv (ATCC 27294), *Pseudomonas aeruginosa* PAO1 (ATCC 47085) and *Acinetobacter baumannii* strain 2208 (ATCC 19606) were used to assess the sensitivity and specificity of the SEA assay. The vector Plasmid DNA (pMD 19-T vector, Takara, Dalian, China) containing 16S rDNA sequence of *M. pneumoniae* M129 was used as positive control. A total of 224 sputum specimens from patients with upper respiratory tract infection were collected from the Affiliated Hospital of Qingdao University during September 2017 and January 2018. All clinical specimens were immediately transported to the laboratory and stored at −20 °C. The authorized Human Health and Ethics Committee of the Affiliated Hospital of Qingdao University approved this study and all the patients were informed consent. In addition, all methods were carried out in accordance with the relevant guidelines and regulations.

### Nucleic acid extraction

Genomic DNA from sputum samples or reference strains was extracted using High Pure Viral Nucleic Acid extraction kit (Roche Applied Science, Mannheim, Germany). In brief, 200 µL sputum samples were added to 200 µL binding buffer supplemented with poly (A) and 50 µL Proteinase K. After incubation for 10 min at 72 °C, the mixture was transferred to a High Pure filter tube combined with a collection tube. The filter tube was centrifuged at 8 000 × g for 1 min and then 500 µL inhibitor removal buffer was added. After centrifugation at 8 000 × g for 1 min, the filter was washed twice with 450 µL washing buffer (20 mM NaCl and 2 mM Tris-HCl [pH 7.5] in ethanol). Finally, the pellet of crude nucleic acids was obtained by centrifugation at 12 000 × g for 1 min, and suspended in 50 µL elution buffer.

### Primers design

SEA and LAMP primers (Table 2) specific to 16S rDNA gene of *M*. *pneumoniae* were designed and evaluated using the NUPACK web tool (http://www.nupack.org/) and the DNAMelt Web Server (http://unafold.rna.albany.edu/?q=DINAMelt). The primers were synthesized by Sangon Biotech (Shanghai, China) and purified by high-performance liquid chromatography (HPLC).

**Table 2:** Primers used for SEA, LAMP and real-time PCR reactions in this work.

Reaction	Primer name	Sequence (5′-3′)	Product length	Target gene
SEA	P1	GCAAGGGTTCGTTATTTGATG	42 bp	16S rDNA
	P2	CTAGCTGATATGGCGCAC		
LAMP	F3	CGCACTTCCTTTGCGTATCAACG	226 bp	
	B3	GGTGTTGGCAATCATGGTTGATCAT		
	FIP	GACCAGGTTCAACGGGAACAGCATCTACTGGTTTACCGATGGTCCAATTGCAGCAG	168 bp	CARDS toxin gene
	BIP	ACCCGGCTGGTCGTGTTGTAGGGGTTTGCAGCTCTTGATAATGAGGGTTG		
	LF	GTCAAGCACTACGGACATTAG	110 bp	
	LB	CTAGAATTAATGAACCGGAAATGC		
Real-time PCR	R1	CCATAACTTTGCCAAGGATGTCA	200 bp	16S rDNA
	F1	CGTAAACGATAGATACTAGCTGTC		

Primers were designed based on 16S rDNA sequence of *M*. *pneumoniae* M129, of which gene bank number is CP017343.1.

### SEA reaction

The SEA reaction was performed using the SEA detection kit and colormetric kit purchased from Navid Biotechnology Co., Ltd. (Qingdao, China). Briefly, the reaction was performed in a 10 µL mixture containing 1 µL of template, 1.5 × 10^−6^ M of primer P1 and P2, 5 µL of 2 × reaction mix, and 0.5 × Eva Green. The reaction mixture was incubated at 65 °C for 60 min, and SEA amplifications were monitored by CFX Connect™ Real-Time PCR System (Bio-Rad, CA, USA) at 1-min intervals. Alternatively, amplification products could be confirmed by the pre-addition of a pH sensitive dye and the color change could be directly observed by the naked eyes.

### LAMP reaction

LAMP reactions were performed in a 10 µL volume consisting of 1 µL template, 1.6 U *Bst* 2.0 DNA polymerase, 2 mM MgSO_4_, 1.6 mM dNTPs, 4.68 µL ddH_2_O and six primers with a final concentration of 0.2 µM F3 and B3, 1.6 µM FIP and BIP, and 0.8 µM LF and LB. The mixture was incubated at 65 °C for 70 min in a CFX Connect™ Real-Time PCR System (Bio-Rad, CA, USA).

### Real-time PCR

Real-time PCR was carried out on a CFX Connect™ Real-Time PCR System (Bio-Rad, CA, USA). Positive and negative controls as well as a no template control (NTC) were included in each run. The reaction procedure was 94 °C for 5 min, followed by 45 cycles of 94 °C for 15 s and 56 °C for 30 s, and terminated by a subsequent cooling step at 25 °C for 10 s.

### Statistical analysis

Each assay was conducted in triplicates and the results were presented by the mean of three measurements. All calculations were performed with SPSS ver. 14.0 (SPSS Inc., Chicago, IL, USA). Comparisons between different detection methods were performed using the Chi-square test or Fisher’s exact test for categorical variables and the ManneWhitney U test for continuous variables. A P value of < 0.05 was considered statistically significant.

## Electronic supplementary material

Rapid diagnosis of mycoplasma pneumonia infection by denaturation bubble-mediated strand exchange amplification: comparison with LAMP and real-time PCR
