# Supplementary material for: Rapid diagnosis of Mycoplasma pneumonia infection by denaturation bubble-mediated strand exchange amplification: comparison with LAMP and real-time PCR
Source: Sci Rep. 2019 Jan 29;9:896. doi: 10.1038/s41598-018-36751-z (PMC6351683; doi:10.1038/s41598-018-36751-z)
Supplement: Supplementary file 1 — Rapid diagnosis of mycoplasma pneumonia infection by denaturation bubble-mediated strand exchange amplification: comparison with LAMP and real-time PCR [file 41598_2018_36751_MOESM1_ESM.docx]

Rapid diagnosis of *mycoplasma pneumonia* infection by denaturation bubble-mediated strand exchange amplification: comparison with LAMP and real-time PCR

Wenqiang Shi^1, #^, Manman Wei^2, #^, Qing Wang^3^, Hongwei Wang^3^, Cuiping Ma^2^ and Chao Shi ^1, *^

**^1^***College of Life Sciences, Qingdao University, Qingdao, 266071, PR China.*

**^2^***Shandong Provincial Key Laboratory of Biochemical Engineering*, *College of Marine Science and Biological Engineering, Qingdao University of Science and Technology, Qingdao, 266042, PR China.*

**^3^***The Laboratory Department of the Affiliated Hospital of Qingdao University, 266101, PR china*


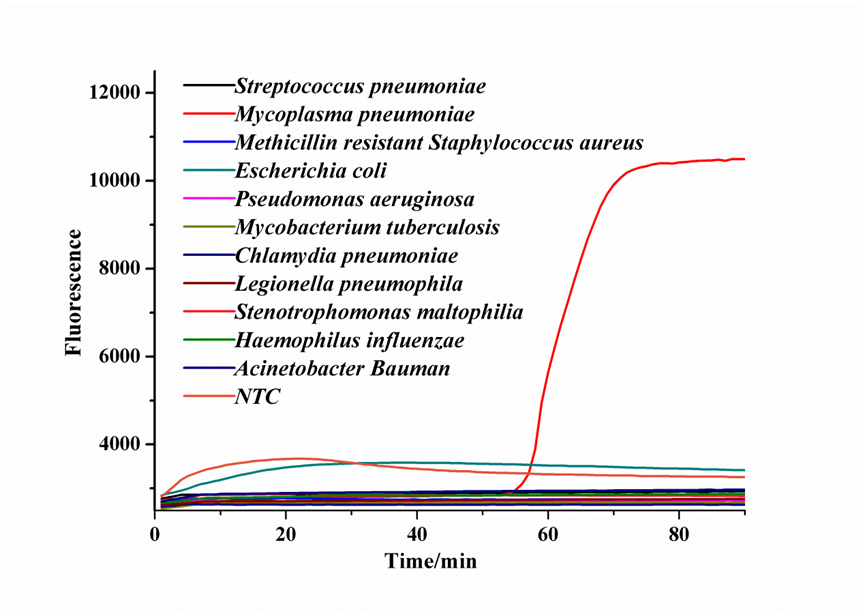


Fig. S1. The real-time fluorescence curves of 11 reference strains including respiratory near neighbors and other pathogens by SEA assay.


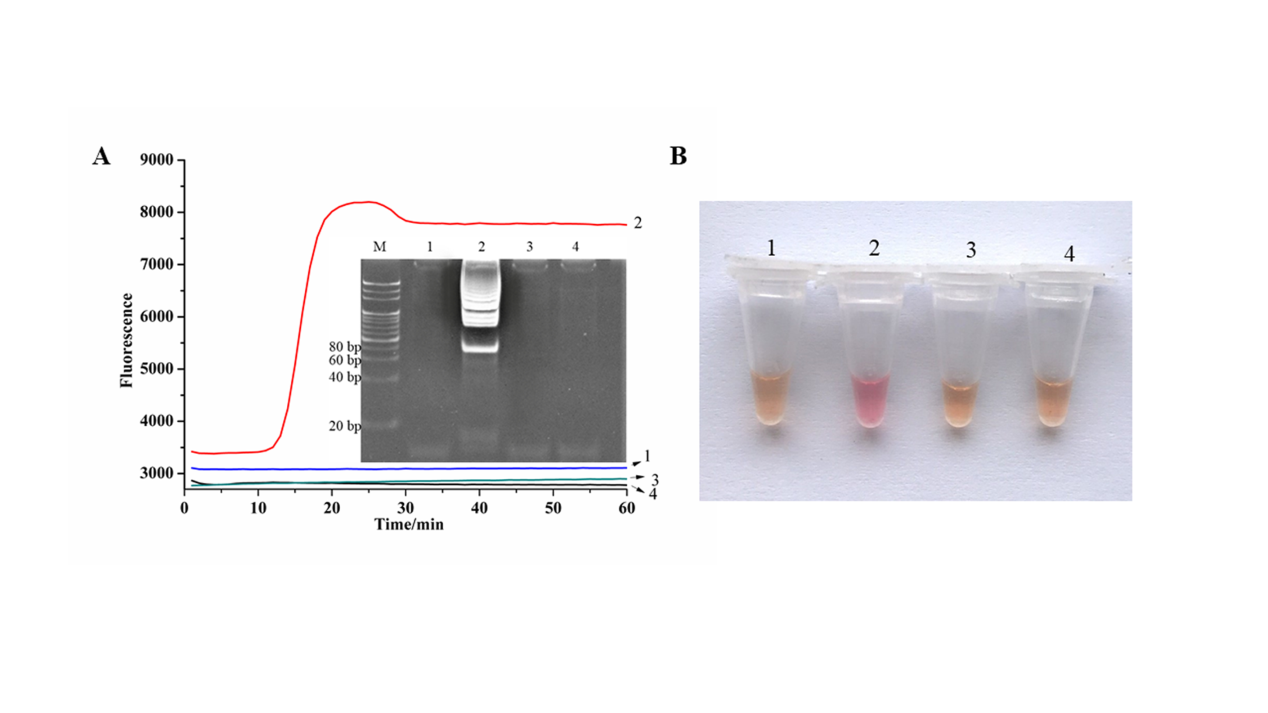


Fig. S2. The sputum specimen with LAMP positive but SEA negative result. (A) The real-time fluorescence curves of SEA reaction (1), LAMP (2) and their respective NTC (3 and 4). Inset: Native PAGE of the corresponding amplification products. (B) Colorimetric assay using neutral red as the indicator.


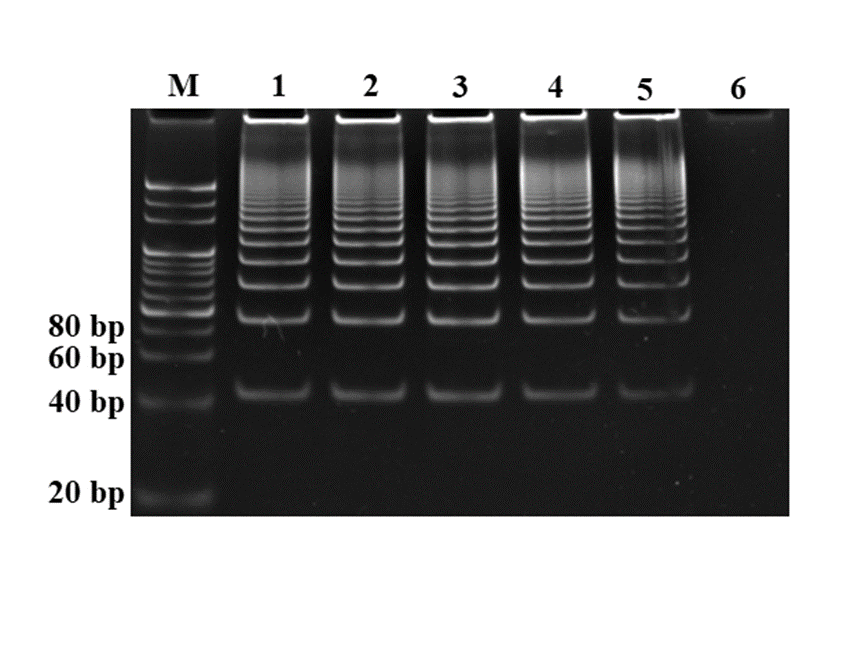


Fig. S3. Native PAGE of the corresponding amplification products.(Lane 1- vector plasmid DNA, Lane 2,3,4,5- genomic DNA extracted from sputum specimen infected by M. pneumoniae , Lane 6-No target control). Fig. 2(B) was constructed with lane M, 1, 2, 6 in this figure.


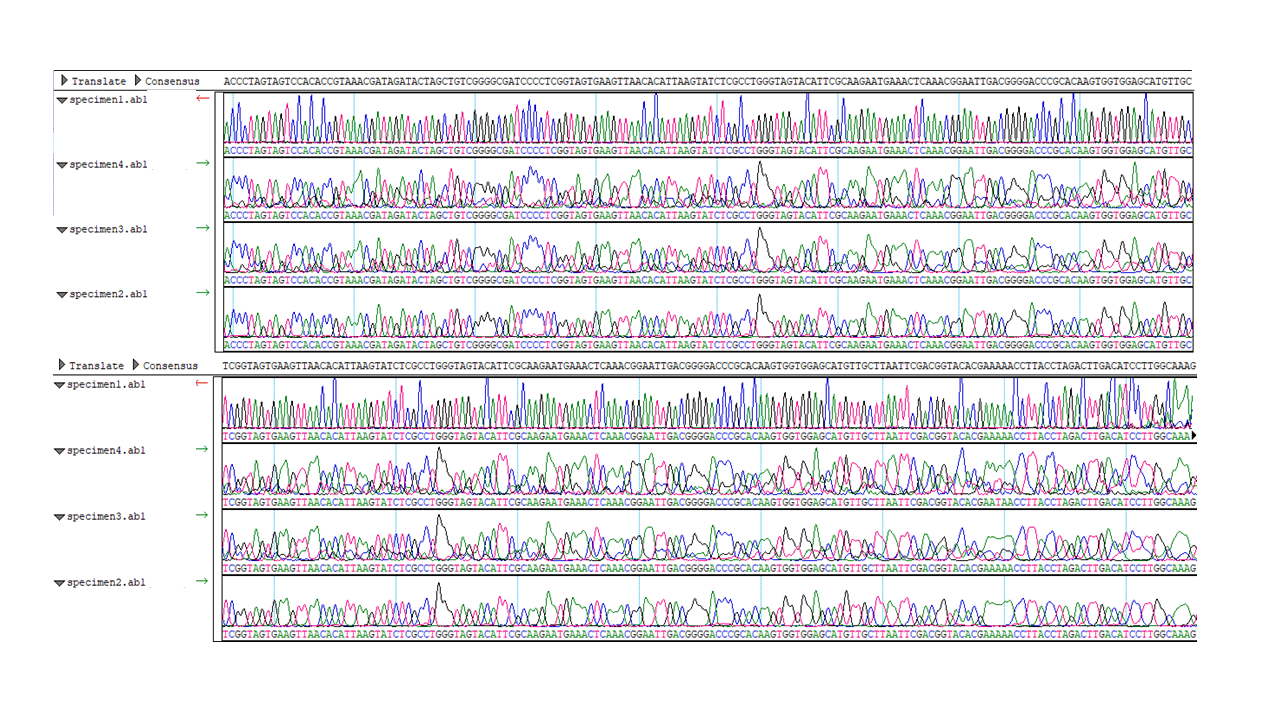


Fig. S4. Sequencing chromatographs of the real-time PCR positive products but negative results for SEA assay and sequences were aligned with reference strain *M. pneumoniae* M129.


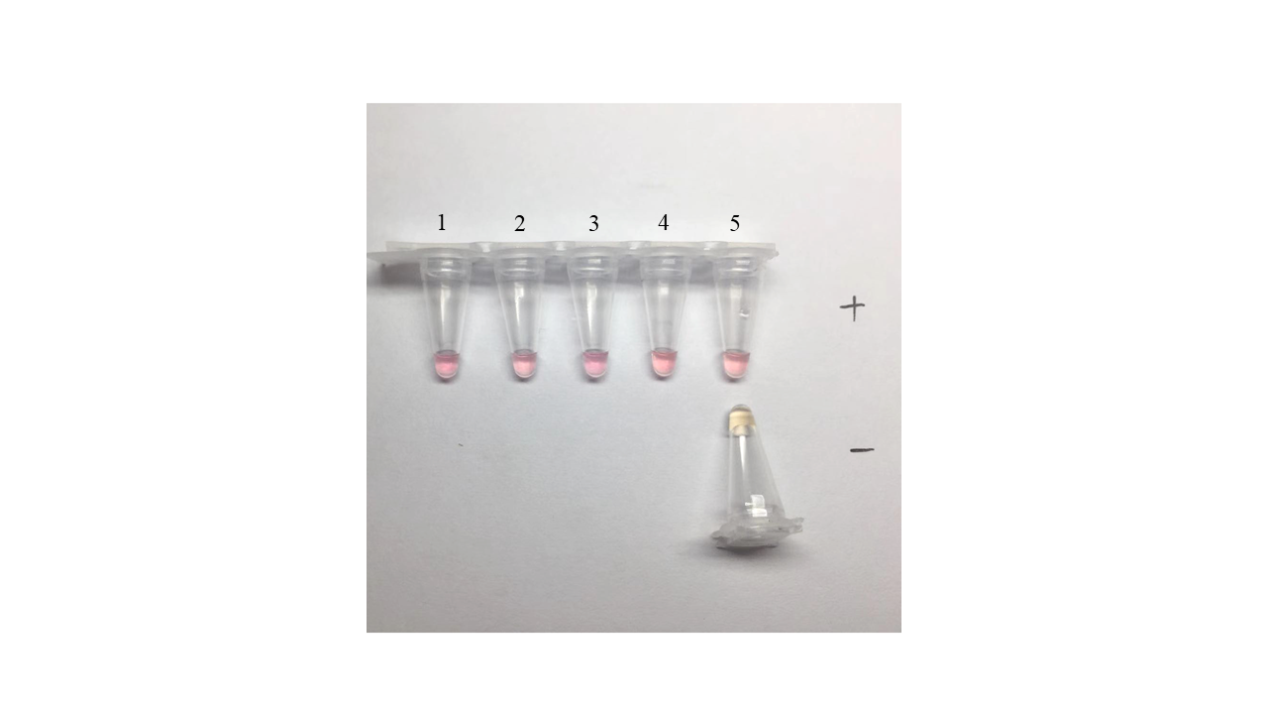


Fig. S5. Colorimetric SEA results with target sequence concentration of 1.0 × 10^8^ (1), 1.0 × 10^7^ (2), 1.0 × 10^6^ (3), 1.0 × 10^5^ (4), 1.0 × 10^4^ (5) and 0 (6) copies/mL.
